# Leukocyte telomere length and lung function: a mendelian randomization study in European population

**DOI:** 10.3389/fphys.2024.1373064

**Published:** 2024-10-24

**Authors:** Shenyu Zhu, Wenlong Zheng, Dingyu Rao, Zhixian Tang, Xinhui Liao

**Affiliations:** ^1^ Department of Thoracic Surgery, The First Affiliated Hospital of Gannan Medical University, Ganzhou, China; ^2^ Cardiothoracic Surgery Brain injury and brain protection key laboratory of Ganzhou, Jiangxi, China; ^3^ Department of Respiratory, Shangyou Hospital of Traditional Chinese Medicine, Ganzhou, China; ^4^ Department of Respiratory, The First Affiliated Hospital of Gannan Medical University, Ganzhou, China

**Keywords:** leukocyte telomere length, lung function, FEV1, mendelian randomization, FVC

## Abstract

**Background:**

The telomere has long been regarded as a dependable biomarker for cellular senescence. The lung function can reflect the function and status of the lungs. As individuals age beyond adulthood, there is a gradual decline in lung function. However, the existence of a associated between leukocyte telomere length (LTL) and lung function remains uncertain.

**Methods:**

A two-sample Mendelian randomization (MR) analysis was used. The Single-nucleotide polymorphisms (SNPs) of LTL from the genome-wide association (GWAS) study were used as exposure instruments variable, and the lung function indicator including Forced expiratory volume in 1-s (FEV1), FEV1 Best measure, FEV1 predicted and Forced vital capacity (FVC) from the Neale Lab and MRC-IEU were used as outcomes. The associated between the exposures and outcomes was assessed using inverse-variance weighted (IVW), MR-Egger, and weighted median methods. Sensitivity analysis was conducted using Cochran’s Q-test, MR-Egger intercept test, MR-PRESSO, leave-one-out analysis, and Steriger test.

**Results:**

Using the IVW method, a significant association was identified between genetically determined telomere length extension and enhanced lung function in FEV1, with ukb-a-336 (P = 0.127, OR = 1.028,95CI% = 1.003–1.042) and ukb-b-19657 (P = 7.26E-05, OR = 1.051,95CI% = 1.025–1.077),in FEV1 predicted, ukb-a-234 (P = 0.013, OR = 1.029,95CI% = 1.003–1.042), ukb-b-8428 (P = 0.001, OR = 1.032,95CI% = 1.012–1.052), in FEV1 best measure, ukb-a-231 (P = 7.24E-05, OR = 1.050,95CI% = 1.025–1.075), ukb-b-11141 (P = 1.40E-09, OR = 1.067,95CI% = 1.045–1.090).The sensitivity analysis did not reveal heterogeneity or horizontal pleiotropy.Meanwhile, the Steriger test results also indicate that the directionality between exposure and outcome is correct. Therefore, the results indicated robustness.

**Conclusion:**

There is a correlation between longer LTL and better lung function in the European dataset.

## Introduction

The telomere, which is a segment located at the terminal end of a chromosome, plays a crucial role in safeguarding genes from harm, thereby ensuring the stability of chromosomes and the integrity of cells (Blackburn, 2001). Telomeres possess significant biological functions, including the stabilization of chromosome functioning, prevention of degradation and fusion at the terminus, protection of chromosome DNA, and regulation of normal cellular processes (Blackburn, 1991). For quite some time, telomeres have been regarded as a dependable biomarker for cellular senescence (López-Otín et al., 2013). In recent years, aside from their correlation with certain age-related diseases (Sanford et al., 2020; Armanios and Blackburn, 2012), telomere shortening has also been linked to an elevated risk of diabetes (Zee et al., 2010) and tumor development (Rode et al., 2016).

The assessment of pulmonary function can serve as an indicator of the respiratory system’s condition in patients. By conducting lung function examinations, healthcare professionals can not only assess the severity of lung diseases and develop targeted treatment strategies but also aid in the early detection of lung and respiratory diseases. A corresponding study has indicated a correlation between prematurely shortened LTL and the presence of idiopathic pulmonary fibrosis (IPF) and chronic obstructive pulmonary disease (COPD) (Dai et al., 2015). The decline of lung function is intricately linked to various lung diseases such as COPD, IPF, and emphysema. Recent studies have indicated a connection between telomere biology and the development of several lung diseases, including IPF and COPD (Alder et al., 2008; Duckworth et al., 2021). However, there is currently a lack of research investigating the potential involvement of LTL in the progression of lung-related diseases through their impact on lung function.

Mendelian randomization (MR) serves as a viable approach to address the common issues of unmeasured confounding and reverse correlation encountered in conventional observational epidemiology (Smith and Ebrahim, 2003). In the present investigation, we employed a dual sample MR design to assess the plausible correlation between LTL and lung function.

## Methods

### Date source

The summary of LTL GWAS results was obtained in the genome-wide meta-analysis, which included 472174 European populations, including 45.8% of males and 54.2% of females (Codd et al., 2021). In the relevant evaluation indicators of lung function, FEV1, FEV1 Best measure, FEV1 predicted and FVCwere selected as the evaluation indicators. For each indicator was driven by two different research consortiums, Neale Lab and MRC-IEU. The dataset ukb-b-19657 of FEV1 includes 421986 cases, and ukb-a-337 includes 307638 cases In FEV1 Best measure, ukb-b-11141 contains 345665 cases, ukb-a-231 contains 255492 cases, ukb-b-8428 in FEV1 predicted contains 148653 cases, ukb-a-234 contains 110423 cases, and in FVC, ukb-a-336 contains 307638 cases., Ukb-b-7953 contains 421986 cases, all of which are from European men and women.

In our meta-regression analysis, we utilized publicly accessible summary data derived from the open GWAS database pertaining to telomere length and lung function datasets. It is worth noting that moral voting was not deemed relevant or applicable to our study.

### Selection of instrumental variables

To be selected as instrumental variables for screening LTL genetic variants, SNPs had to meet three key assumptions: 1) strong association with exposure factors, 2) no association with any confounders of exposure factor-outcome associations (horizontal pleiotropy), and 3) the genetic variant only affects the outcome through exposure factors and not through other pathways.In the process of instrumental variables (IV) selection, the initial criterion employed is whole genome significance (*p* < 5 × 10^−8^). Additionally, to eliminate the presence of Linkage disequilibrium, R^2^ < 0.001 and a window size of 10000 kb are set. Consequently, a total of 154 SNPs are ultimately identified as IVs for telomere length. It is noteworthy that the F-statistic for all these genetic variations surpasses the threshold of 10.

### Mendelian randomization analysis

In the course of the analysis, the IVW method served as the primary approach for assessing the correlation between LTL and lung function. Additionally, the MR Egger, Weighted median, Simple mode, and Weighted mode methods were employed for validation purposes. The evaluation of IVs’ heterogeneity was conducted through the utilization of MR Egger regression (MR Egger intercept test) and IVW in Cochran’s Q statistic (Greco et al., 2015). A significance level of *p* > 0.05 was employed to determine the absence of heterogeneity or horizontal pleiotropy. Simultaneously, we employed the Mendelian randomization Randomized Multivalidity Residual and Outlier (MR-PRESSO) test to detect and eliminate outliers at the level (Verbanck et al., 2018). Throughout this process, we utilized retention analysis to mitigate the potential influence of a single SNP on the overall findings. Furthermore, we employed the Steiger test to compute the variance explained by the instrumental variables estimation on both the exposure and outcome variables, and to determine if the outcome’s variance (c) is indeed lower than that of the exposure. If this condition is met, the direction is deemed accurate (Hemani et al., 2017).

In this study, statistical analyses were performed using R code (version 4.2.2). MR analyses were conducted using the “TwoSampleMR” package (version 0.5.6) (Hemani et al., 2018)and the “MR-PRESSO” package (version 0.5.6).

## Results

Based on the results of the MR analysis, a potential correlation was observed between extended LTL and better lung function indicators, FEV1, predicted FEV1, the best measure of FEV1, and FVC. Employing the IVW method, a significant correlation was identified between genetically determined LTL extension and enhanced lung function in FEV1, with ukb-a-336 (P = 0.127, OR = 1.028,95CI% = 1.003–1.042) and ukb-b-19657 (P = 7.26E-05, OR = 1.051,95CI% = 1.025–1.077), in FEV1 predicted, ukb-a-234 (P = 0.013, OR = 1.029,95CI% = 1.003–1.042), ukb-b-8428 (P = 0.001, OR = 1.032,95CI% = 1.012–1.052), in FEV1 best measure, ukb-a-231 (P = 7.24E-05, OR = 1.050,95CI% = 1.025–1.075), ukb-b-11141 (P = 1.40E-09, OR = 1.067,95CI% = 1.045–1.090), in FVC, ukb-a-336 (P = 0.024, OR = 1.022,95CI% = 1.003–1.042), ukb-b-7953 (P = 0.038, OR = 1.018,95CI% = 1.001–1.036) (Table 1).

**TABLE 1:** Genetic prediction of telomere length (TL) and its association with lung function indicators (FEV1,FEV1 predicted,FEV1 best measure and FVC.

Eposure	Otcome		method	nsnp	OR (95%CI)	pval
leukocyte telomere length	FEV1	ukb-b-19657	MR Egger	51	1.062 (1.016–1.109)	0.010
			Weighted median		1.058 (1.021–1.097)	0.002
			Inverse variance weighted		1.051 (1.025–1.077)	7.26E-05
			Simple mode		0.984 (0.907–1.068)	0.706
			Weighted mode		1.064 (1.025–1.104)	0.002
		ukb-a-337	MR Egger	115	1.035 (0.976–1.097)	0.257
			Weighted median		1.051 (1.019–1.083)	0.002
			Inverse variance weighted		1.056 (1.020–1.093)	0.002
			Simple mode		1.037 (0.981–1.096)	0.199
			Weighted mode		1.047 (1.017–1.077)	0.002
	FEV1 predicted	ukb-b-8428	MR Egger	108	1.032 (0.999–1.067)	0.061
			Weighted median		1.03 (0.998–1.063)	0.068
			Inverse variance weighted		1.032 (1.012–1.052)	0.001
			Simple mode		1.06 (0.998–1.126)	0.061
			Weighted mode		1.031 (1–1.062)	0.052
		ukb-a-234	MR Egger	111	1.051 (1.012–1.092)	0.012
			Weighted median		1.042 (1.005–1.082)	0.027
			Inverse variance weighted		1.029 (1.006–1.052)	0.013
			Simple mode		1.049 (0.967–1.137)	0.252
			Weighted mode		1.039 (0.998–1.081)	0.064
	FEV1 best measure	ukb-b-11141	MR Egger	69	1.072 (1.033–1.111)	0.000
			Weighted median		1.068 (1.037–1.1)	1.45E-05
			Inverse variance weighted		1.067 (1.045–1.09)	1.40E-09
			Simple mode		1.063 (1–1.131)	0.056
			Weighted mode		1.071 (1.039–1.103)	3.79E-05
		ukb-a-231	MR Egger	76	1.047 (1.005–1.091)	0.030
			Weighted median		1.036 (0.997–1.077)	0.070
			Inverse variance weighted		1.05 (1.025–1.075)	7.24E-05
			Simple mode		1.077 (0.995–1.166)	0.072
			Weighted mode		1.046 (1–1.093)	0.053
	FVC	ukb-b-7953	MR Egger	89	1.025 (0.996–1.055)	0.100
			Weighted median		1.043 (1.017–1.07)	0.001
			Inverse variance weighted		1.018 (1.001–1.036)	0.038
			Simple mode		1.023 (0.965–1.084)	0.446
			Weighted mode		1.045 (1.019–1.072)	0.001
		ukb-a-336	MR Egger	94	1.034 (1.001–1.068)	0.046
			Weighted median		1.052 (1.02–1.084)	0.001
			Inverse variance weighted		1.022 (1.003–1.042)	0.024
			Simple mode		1.028 (0.953–1.11)	0.477
			Weighted mode		1.06 (1.024–1.097)	0.001

FEV1, Forced expiratory volume in 1-s; FVC, Forced vital capacity; SNPs, single nucleotide polymorphisms; IVW, inverse variance weighted (random-effects model); IVW, inverse variance weighted (fixed-effects model); MR-Egger, Mendelian randomization-Egger; OR, odds ratio; CI, confidence interval.

**FIGURE 1:**
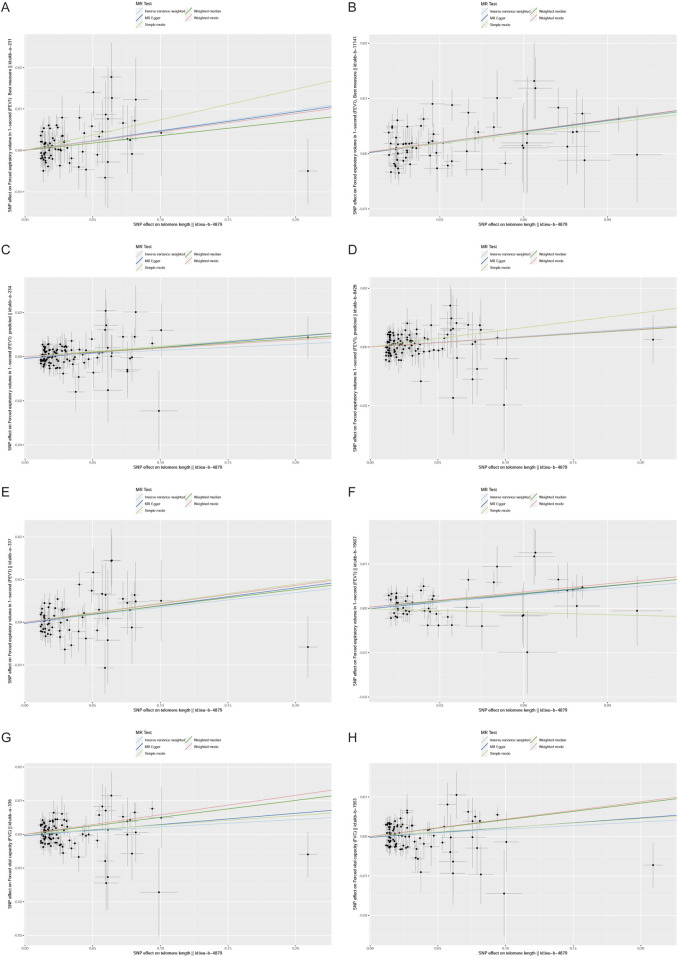
Scatter plot of the association between the telomere length (TL) and lung function indicators (FEV1,FEV1 predicted,FEV1 best measure and FVC).

Furthermore, we employed various methods to assess the results obtained from the IVW method, all of which yielded consistent directions and similar effects (Figure 1). Our analysis of FVC outcomes using Cochran’s Q test and MR Egger test revealed varying degrees of heterogeneity (Table 2). Additionally, we conducted statistical analysis using the intercept term egger of MR egger_ intercept, which yielded a *p*-value greater than 0.05, indicating the absence of pleiotropy (Table 2). However, we conducted a retest of pleiotropy levels using MR-PRESSO and identified the presence of pleiotropy in FVC-related outcomes (ukb-a-336, P = 0.031, ukb-b-7953, P = 0.0016).

**TABLE 2:** Heterogenity test and Horizontal pleiotropy test of telomere length (TL) and its association with lung function indicators (FEV1,FEV1 predicted,FEV1 best measure and FVC.

Exposure	Outcome	Heterogenity test				Horizontal pleiotropy test			
		Method	Q	Q_df	Q_pval	Egger_Intercept	se	Pval	Mrpresso
leukocyte telomere length	ukb-a-337 FEV1	MR Egger	81.883	68	0.120	−2.60E-04	5.98E-04	0.665	0.121
		IVW	82.111	69	0.134				
	ukb-b-19657 FEV1	MR Egger	66.393	49	0.050	−3.64E-04	6.55E-04	0.581	0.063
		IVW	66.811	50	0.056				
	ukb-a-234 FEV1 predicted	MR Egger	104.985	109	0.591	−7.92E-04	5.78E-04	0.173	0.577
		IVW	106.863	110	0.567				
	ukb-b-8428 FEV1 predicted	MR Egger	98.632	106	0.682	−6.47E-06	4.95E-04	0.990	0.725
		IVW	98.632	107	0.706				
	ukb-a-231 FEV1 Best measure	MR Egger	85.817	74	0.164	7.87E-05	6.17E-04	0.899	0.186
		IVW	85.836	75	0.184				
	ukb-b-11141 FEV1 Best measure	MR Egger	87.407	67	0.048	−1.50E-04	5.84E-04	0.797	0.071
		IVW	87.494	68	0.056				
	ukb-a-336 FVC	MR Egger	115.995	92	0.046	−4.17E-04	4.89E-04	0.396	0.031
		IVW	116.911	93	0.047				
	ukb-b-7953 FVC	MR Egger	125.922	87	0.004	−2.45E-04	4.56E-04	0.592	0.0016
		IVW	126.340	88	0.005				

**FIGURE 2:**
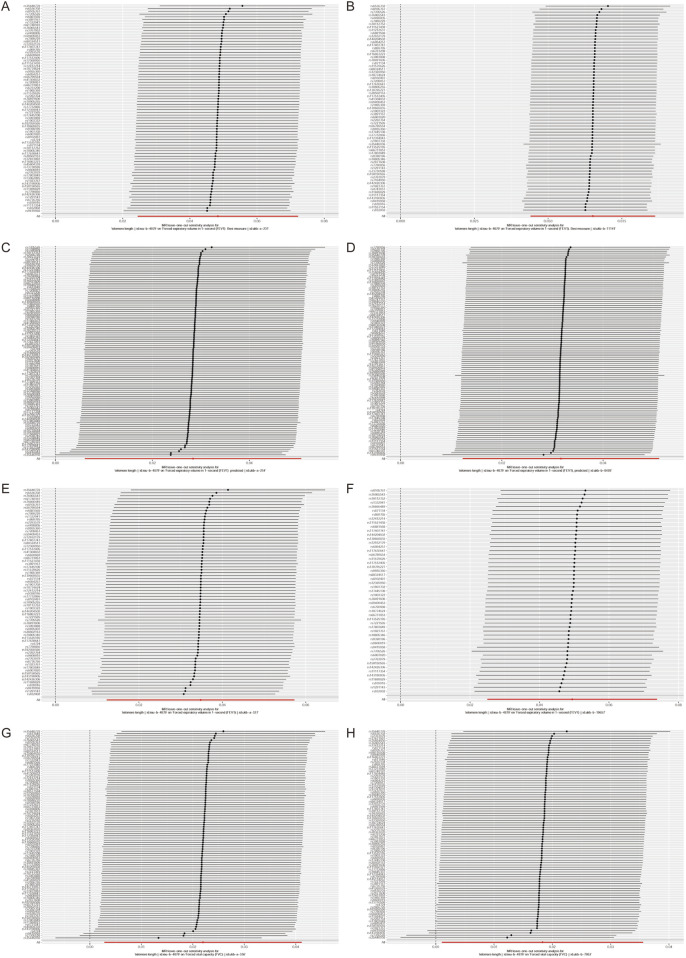
Leave‐one‐out analyses to evaluate whether any single instrumental variable was driving the correlation effect.

In the context of sensitivity analysis, the exclusion of a single SNP does not significantly alter the correlation estimation of genetic predicted LTL on lung function (Figure 2). This implies that the removal of any specific SNP does not fundamentally impact the results, thereby ensuring the stability of our dual sample MR analysis. Additionally, the Steriger test reveals that the variance explained by instrumental variables estimation in the outcome is lower than that in the exposure. This suggests that the instrumental variables estimation influences the outcome primarily through its effect on the exposure, as supported by the data presented in Supplementary Table S1.

## Discussion

In this randomized study utilizing a two-sample MR approach, our findings indicate a positive association between longer LTL and improved lung function indicators, encompassing FEV1, predicted FEV1, and the most accurate measure of FEV1. Consequently, acquiring an in-depth comprehension of the interplay between LTL and lung function holds immense importance in the realm of lung disease prevention and treatment.

LTL functions as a crucial indicator reflecting the cellular biological condition and overall health status. The shortening of LTL is associated with an augmented risk of cardiovascular diseases, diabetes, and certain cancers (Pauleck et al., 2023; Haycock et al., 2014; Ahmed and Lingner, 2018). Relevant studies have demonstrated that neutrophils induce telomere dysfunction through reactive oxygen species (ROS) dependence (Lagnado et al., 2021). Simultaneously, LTL is prone to ROS-induced impairment, and oxidation can result in telomere shortening (Ahmed and Lingner, 2018). Nevertheless, the length of LTL can be maintained via the UFMylation of MRE11 (Lee et al., 2021). The regulatory mechanism of LTL encompasses multiple aspects such as telomerase activity, oxidative stress, inflammatory response, and genetic factors, yet the specific one remains ambiguous. LTL is a vital regulator of Cellular senescence and genome stability. The deterioration of lung function indicates the aging process. Research indicates that individuals aged 25 to 40 with decreased lung function (FEV<80% predicted) in early adulthood have a higher risk of developing respiratory and cardiovascular diseases, as well as a greater all-cause mortality rate (Agustí et al., 2017).Numerous studies have verified the correlation between LTL and the lung disease. Some research disclosed that individuals with shorter telomere length presented reduced lung function and an elevated risk of developing COPD (Rode et al., 2013; Savale et al., 2009). At the same time, forced expiratory volume (FEV) decreased significantly more in smokers with shorter LTL than in smokers with longer LTL (Andujar et al., 2018). Alder et al. conducted research on the role of telomere length as a genetic predisposing factor for Emphysema by inducing a chronic cigarette smoke model (Alder et al., 2011). Both LTL and lung function decline with age. At the same time, there was a moderate correlation between shorter telomere length in middle-aged people and compromised airflow parameters, although there was no significant correlation with FVC, which is consistent with our own findings (Nguyen et al., 2019). Studies have shown that telomere shortening has been identified as a characteristic of idiopathic interstitial pneumonia and may lead to idiopathic sexual organ failure, which is clinically manifested by lung and liver problems (Alder et al., 2008; Duckworth et al., 2021). Additionally, it has been observed that individuals with shorter telomere length have an increased risk of death and a poorer prognosis among lung cancer patients (Zhang et al., 2015; Jang et al., 2008; Doherty et al., 2018; Astuti et al., 2017).

It should be emphasized that telomere length can be affected by various genetic and environmental factors, including age, lifestyle choices, psychological factors, and the existence of other disease conditions. Numerous studies have indicated that although there is currently a lack of direct evidence to prove that exercise can prolong LTL, a significant difference in LTL is observed between regular exercisers and non-exercisers, as demonstrated by comparative analyses of exercise and non-exercise groups (Cherkas et al., 2008; Venturelli et al., 2014; Borghini et al., 2015; Silva et al., 2016). Hence, adopting a healthier lifestyle and avoiding smoking may contribute to maintaining longer telomere length and optimal lung function.

In this study, there are indeed certain limitations. Our investigation was solely centered on the relationship between LTL and lung function. While we recognize that other factors such as genetic variations and environmental influences may also play a role in lung function. Additionally, it should be noted that our study only included individuals of European descent. Hence, future data from other populations are required to verify this result.

## Conclusion

Our research findings indicate a correlation between increased LTL and enhanced lung function. Integrating LTL measurement into clinical practice holds promise for valuable insights into individual risk assessment, disease prognosis, and potential treatment approaches. Further investigation is warranted to elucidate the underlying mechanisms and assess the potential of LTL-related interventions in ameliorating lung function and health.

## Data Availability

The original contributions presented in the study are included in the article/Supplementary Material, further inquiries can be directed to the corresponding authors.
