# Supplementary material for: Leukocyte telomere length and lung function: a mendelian randomization study in European population
Source: Front Physiol. 2024 Oct 24;15:1373064. doi: 10.3389/fphys.2024.1373064 (PMC11540648; doi:10.3389/fphys.2024.1373064)
Supplement: Supplementary file 1 [file Table1.DOCX]

| **Exposure** | **Outcome** | **snp_r2.exposure** | **snp_r2.outcome** | **correct_causal_direction** | **steiger_pval** |
| --- | --- | --- | --- | --- | --- |
| **leukocyte telomere length** | ukb-a-337  FEV1 | 0.022243806 | 0.000372916 | TRUE | 0 |
|  | ukb-a-234  FEV1 predicted | 0.028284397 | 0.001145785 | TRUE | 0 |
|  | ukb-a-336  FVC | 0.025118301 | 0.000448549 | TRUE | 0 |
|  | ukb-a-337  FEV1 | 0.022243806 | 0.000372916 | TRUE | 0 |
|  | ukb-b-7953  FVC | 0.024613947 | 0.000370471 | TRUE | 0 |
|  | ukb-b-8428  FEV1 predicted | 0.027348145 | 0.000840025 | TRUE | 0 |
|  | ukb-b-11141  FEV1 Best measure | 0.021844275 | 0.000505825 | TRUE | 0 |

**Supplement table 1** Steiger test of telomere length (TL) and lung function indicators（FEV1,FEV1 predicted,FEV1 best measure and FVC.
